# Association of *CYP1A1, GSTM1* and *GSTT1* gene polymorphisms with risk of non-small cell lung cancer in Andhra Pradesh region of South India

**DOI:** 10.1186/s40001-016-0209-x

**Published:** 2016-04-18

**Authors:** Vidyullatha Peddireddy, Siva Prasad Badabagni, Sandhya Devi Gundimeda, Vasudha Mamidipudi, Pardhanandana Reddy Penagaluru, Hema Prasad Mundluru

**Affiliations:** Institute of Genetics and Hospital for Genetic Diseases, Osmania University, Begumpet, Hyderabad, 500016 Telangana India; Indo-American Cancer Hospital, Banjara Hills, Hyderabad, 500034 Telangana India; Bhagwan Mahavir Medical Research Centre, Hyderabad, 500004 Telangana India; DST Woman Scientist, Department of Biotechnology, University of Hyderabad, Gachibowli, Hyderabad, 500046 Telangana India

## Abstract

**Background:**

Lung cancer is one of the most preventable causes of death globally both in developed and developing countries. Although it is well established that smokers develop lung cancer, there are some smokers who are free from the disease risk. The predisposition to lung cancer is attributed to genetic polymorphisms in xenobiotic metabolizing genes. Reports on assessment of xenobiotic metabolizing genes like *Cytochrome P 450 1A1* (*CYP1A1), Glutathione* -*S* -*transferase M1 (GSTM1)* and T1 (*GSTT1)* polymorphisms from India are meagre, and reports from Andhra Pradesh are lacking.

**Methods and results:**

Assessment of polymorphisms in *CYP1A1, GSTM1* and *GSTT1* in NSCLC patients and healthy individuals specific to population of Andhra Pradesh, a South Indian state was attempted by multiplex PCR and RFLP, and this is the first study which tried to correlate oxidative stress with the polymorphisms in xenobiotic metabolizing genes. Results showed that *CYP1A1 m1* ‘CC’ genotype was significantly associated with lung cancer susceptibility with a 2.3-fold risk, *CYP1A1 m2* ‘AG’ gene polymorphisms with 8.8-fold risk and *GSTT1* (−/−) genotype demonstrated a twofold risk of disease susceptibility.

**Conclusions:**

A combined role of genetic polymorphisms and smoking status can be attributed for the cause of lung cancer. Further, the association between oxidative stress and genetic polymorphisms showed a correlation between *GSTT1* and super oxide dismutase activity; *CYP1A1 m1*, *m2* and *GSTT1* with glutathione peroxidase activity; *CYP1A1 m1* and *GSTM1* with melondialdehyde levels; and *CYP1A1 m1* and *GSTT1* with 8-oxo-7,8-dihydro-2′-deoxyguanosine. A higher risk of lung cancer seems to be associated with combined gene polymorphisms of phase I and phase II enzymes than that ascribed to single gene polymorphism.

**Electronic supplementary material:**

The online version of this article (doi:10.1186/s40001-016-0209-x) contains supplementary material, which is available to authorized users.

## Background

Xenobiotic metabolism is the process of detoxification of endogenous or exogenous carcinogens/poisons and occurs in two phases. In Phase I, c*ytochrome P450* oxidases amend the xenobiotics by introducing a polar or reactive group. In Phase II, the modified xenobiotics are conjugated to polar compounds facilitated by enzymes such as glutathione S-transferases [1]. Among the phase I enzymes, cytochrome P450 1A1 (CYP1A1) plays a vital role in the activation of polycyclic aromatic hydrocarbons (PAHs) to convert them to carcinogens [2]. The phase II enzymes involve glutathione-S-transferases (GSTs*)* which are divided into five classes (alpha, mu, pi, theta and zeta), and catalyse the conjugation of highly reactive PAHs to soluble glutathiones [3]. Among the GSTs, *GSTM1* preferentially detoxifies carcinogens (epoxides and hydroxylated derivatives) derived from tobacco, whereas *GSTT1* causes the biotransformation of many toxins such as butadiene and ethylene oxides (ingredients of tobacco smoke) [4]. The balance between the phase I and phase II enzymes is crucial to determine the amount of reactive intermediates that are formed in the cell. Any aberrations due to genetic polymorphisms affect the activities of these enzymes; thereby, increasing the risk of cancer in an individual and gene–gene interactions of phase I and phase II enzymes together with life style habits can be synergistic risk factors.

Among all the cancers, carcinoma of the lung is responsible for the high death rate throughout the world [5]. Although tobacco consumption is considered to be the significant aetiological factor for lung cancer [6], not all smokers develop lung cancer. Risk is dependent on the extent of smoking, environmental factors (carcinogen exposure) and most prominently genetic factors. Genetic polymorphisms in the enzymes involved in metabolic activation and detoxification were found to immensely contribute to the risk of developing lung cancer [7]. These polymorphisms cause inter-individual differences in the bio-activation and detoxification of pro-carcinogens, which are in turn responsible for the varied susceptibilities to lung cancer [8, 9]. Among the xenobiotic metabolizing enzymes, *CYP1A1, GSTM1* and *GSTT1* have been projected as the potential modulators of cancer susceptibility [10]. Although these enzymes play a crucial role in bio-activation and detoxification of chemical carcinogens present in tobacco smoke, the role of Glutathione–S transferase genes in modulating the risk of cancer has been debated owing to inter-individual, geographical, ethnic and demographical differences throughout the world. The association between *CYP1A1* and *GSTM1* polymorphisms in lung cancer was reported [11, 12]. However, *GSTT1* deficiency was demonstrated (*GSTT1* null) not to increase the risk of lung cancer [13, 14]. The frequencies of *CYP1A1* and *GSTM1* gene polymorphisms were found to vary among different ethnic populations [15, 16]. Among Asians, *CYP1A1 2A* and *CYP1A1 2C* genetic polymorphisms are common, whereas in Caucasians, the variation in *CYP1A1 2C* is rare [16, 17]. Similarly, *GSTM1* null type is more common in Asians than in Caucasians [18]. Null genotype represents the homozygous deletion of the gene. The inter-relation between *CYP1A1* polymorphism, tobacco smoking and lung cancer was found to be high in Japanese and Chinese populations, whereas the same was not observed in Caucasians [17, 19–22]. The risk association between *GSTM1* null genotype with squamous cell and small cell carcinomas in Asians was found to be significant [23, 24]. Further, a combination of *GSTM1* null genotype with *CYP1A1* polymorphisms augmented lung cancer risk [25, 26].

In the Indian context, studies on association of lung cancer and genetic polymorphisms are limited. In a North Indian cohort, the risk of *CYP1A1* gene polymorphism in 100 patients with lung cancer was assessed, and a 2.68-fold risk was observed for *CYP1A1 2C* allele and in the presence of a single copy of the variant *CYP1A1* (*CYP1A1* * *1/2A*) and for null *GSTT1* genes, a threefold increased risk of lung cancer was demonstrated [27]. Another group from North India demonstrated that the risk of lung cancer is associated with *CYP1B1* and *GSTM1* polymorphisms in the population [28]. *CYP2E1* polymorphisms in six ethnic groups of South Indian population were demonstrated [29]. A study from Kerala in 146 lung cancer patients indicated that *CYP1A1 MspI* homozygous variant allele and *GSTT1* null deletion frequency were significantly higher in smoking-induced lung cancer patients compared with other populations [30]. The Southern part of India is largely composed of five states, namely, Andhra Pradesh (Telangana + Andhra Pradesh), Tamilnadu, Kerala, Karnataka and Maharashtra, where the environmental conditions, economy, food habits and life style vary a lot. The limitations of systematic studies that correlate the association between *CYP450* and *GST* gene polymorphisms and the risk of lung cancer include (1) limited number of subjects from different areas which are not representative of the entire population; (2) subjects exposed to different environmental conditions; and (3) different gene polymorphisms being evaluated. Besides ethnic background, life style and dietary habits also contribute to the increased risk of lung cancer. The dietary habits, environmental factors and tobacco consumption vary between the Northern and Southern regions of India. Tobacco consumption is rampant in both North and South Indian populations [31]. Although studies reported the association of lung cancer and gene polymorphisms in South Indian population, the samples were drawn from a tertiary hospital located in the capital city of a particular state. Hence, the entire South Indian population was not represented. In view of the above, it becomes imperative to determine the association between the gene polymorphisms of enzymes that are associated with detoxification of tobacco-related carcinogens and the risk of lung cancer in the state of Andhra Pradesh to generate more data to arrive at a plausible conclusion. Further, the samples were collected from a single hospital that received patients from the entire state of Andhra Pradesh, and this ensured homogeneity of the samples obtained.

Besides genetic factors, biochemical markers such as oxidative stress and antioxidant responses were also implicated in the development of lung cancer which showed changes in the oxidant and antioxidant statuses in the peripheral lymphocytes of non-small cell lung cancer (NSCLC) patients [32]. However, till date, studies that demonstrate the association between genetic and biochemical interactions and the risk of NSCLC were not reported. Hence, for the first time, in this study, we analysed such associations which might serve as predictive markers, contributing to differential susceptibility toward PAH and tobacco-induced cancers.

## Methods

### Study population

The subjects of the present study included 246 newly diagnosed and previously untreated NSCLC patients referred to Indo-American cancer Hospital from various regions of Andhra Pradesh, India during the period June 2008–2012. 98.2 % of the patients and all the control subjects included in our study were natives of Andhra Pradesh. All the patients were rated as positive for NSCLC by histological analyses and were classified using revised lung cancer staging system [33]. The co-morbid conditions in NSCLC patients included 24 (9.75 %) of them being diabetic, 30 (12.19 %) hypertensive and 2 (0.81 %) patients having hypothyroidism. Age- and sex-matched healthy controls (*n* = 250) were enroled from the general population of the same geographical region. Routine medical check-up was conducted, and history of illness was recorded by a health practitioner. Those who appeared apparently healthy without any history of cancer or other chronic diseases were considered as normal. The co-morbid conditions among controls included 8 (3.2 %) of them being diabetic, 11 (4.4 %) being hypertensive, and none having hypothyroidism. Study subjects who were used to smoking at the time of diagnosis were considered as smokers and those who had smoked at least 100 cigarettes in their life time were considered as ex-smokers. Among the NSCLC smokers, 51 (48.11 %) and 47 (44.34 %) consumed cigarettes and bidis, respectively, and 8 (7.55 %) consumed both. In the case of NSCLC patients who are ex-smokers, the cigarette and bidi consumers were 26 (61.9 %) and 15 (35.72 %), and 1 (2.38 %) consumed both bidi as well as cigarette. Pack years were computed as the number of cigarettes smoked per day multiplied by the duration of smoking in years, and the average tobacco consumption was expressed in pack years. Among the control smokers, 48 (76.19 %) consumed cigarettes, while 15 (23.81 %) were bidi smokers. In case of ex-smokers, 8 (66.66 %) were cigarette smokers, while 4 (33.34 %) were bidi smokers.

### Ethics statement

The study was carried out with the approval of Institutional Ethics Committees of Indo-American Cancer Hospital and Institute of Genetics and Hospital for Genetic Diseases. Educated and informed consent was obtained from all the subjects of the study. A standard questionnaire was used to document the socio-demographical characteristics such as age, sex, lifestyle (alcohol, diet, etc.), occupational exposure (working hours/day, years of exposure, use of protective measures, etc.), history of smoking, number of cigarettes per day and duration of smoking.

### Molecular analysis of *CYP1A1 m1, m2, GSTM1* and *GSTT1* gene polymorphisms

#### Blood collection and DNA isolation

2 ml of whole blood was collected in vacutainers (BD Biosciences) containing ethylenediamine tetra acetic acid (EDTA) for DNA isolation, and 3 ml was collected in heparinized vacutainers for the assessment of oxidative stress markers from healthy controls and NSCLC patients. Genomic DNA was isolated (Flexi gene extraction kit, QIAGEN) from 300 μl of whole blood and was stored in −80 °C until further use.

#### *CYP1A1 m1* and *m2* genotyping

Genotyping for *CYP1A1 m1* and *m2* genes (rs4646903 and rs1048943) was carried out as described earlier [34]. The primers’ sequences used for m1 site were M1F (5′-CAG TGA AGA GGT GTA GCC GCT-3′) and M1R (5′-TAG GAG TCT TGT CTC ATG CCT-3′) and for m2 site were M2F (5′- TTC CAC CCG TTG CAG GAT AGC C-3′) and M2R (5′-CTG TCT CCC TCT GGT TAC AGG AAG-3′). The PCR amplification was carried out in 25-µl reaction mixture consisting of 100 ng template of DNA, 10 µM of each primer, 0.2 mM each dNTP, 2.4 mM MgCl_2_, 1 U Taq DNA polymerase with 1× reaction buffer (Bangalore Genei). The PCR cycle consisted of 1 min at 94 °C, 1 min at 61 °C (for *CYP1A1 m1*)/63 °C (for *CYP1A1* m2) and 1 min at 72 °C with initial denaturation of 5 min at 94 °C and final extension of 10 min at 72 °C. The PCR amplicons generated for m1 (340 bp) and m2 (204 bp) were subjected to restriction digestion. *Msp1* and *BsrD1* restriction enzymes were used to detect polymorphisms in the *CYP1A1 m1* and *m2*, respectively. The reaction mixtures were incubated at 37 °C for 12 h, electrophoresed on 3.0 % agarose gel and stained with ethidium bromide (Sigma Aldrich, USA) for visualization. All the sampling experiments were done in duplicates. Restriction digestion was repeated in cases which were unclear. Positive samples were included in each run of PCR as well as restriction digestion to ensure that the samples were properly digested.

#### *GSTM1* and *GSTT1* genotyping

The *GSTM1* and *GSTT1* gene deletions (rs4025935 and rs71748309) were analysed simultaneously by multiplex PCR [35]. To detect the *GSTM1* deletion, the primers used were *GSTM1* F (5′-GAACTCCCTGAAAAGCTAAAGC-3′) and *GSTM1* R (5′-GTTGGGCTCAAATATACGGTGG-3′). For *GSTT1*, the primers used were *GSTT1* F (5′-TTCCTTACTGGTCCTCACATCTC-3′) and *GSTT1*R (5′-TCACCGGATCATGGCCAGCA-3′). The PCR amplicons were electrophoresed on a 4 % agarose gel, stained with ethidium bromide, and the results were documented using a gel documentation system (Bio-Rad). The presence of *GSTM1* and that of *GSTT1* genes were indicated by the resulting 215- and 480-bp PCR amplicons, respectively. A DNA sample with *GSTM1* and *GSTT1* alleles present was run as a positive control in each run. As an internal control, human albumin gene (*HAB*) was amplified (350 bp) using the primers *HAB* F (5′-CAACTTCATCCACGTTCACC-3′) and *HAB* R (5′-GAAGAGCCAAGGACAG GTAC-3′) for the authentication of multiplex PCR.

### Estimation of 8-oxodG, lipid peroxidation and antioxidant enzymes

8-oxo-7,8-dihydro-2′-deoxyguanosine (8-oxodG) levels in the urine samples of healthy controls and NSCLC patients were measured in 125 patients and 100 controls using commercially available kits (Japan Institute for the Control of Aging, Shizuoka, Japan). Lipid peroxidation products were measured in the plasma of 246 patients and 250 controls as described earlier [32]. Red cell superoxide dismutase (SOD) and glutathione peroxidase (GPx) activities were estimated in 238 NSCLC patients and 250 controls using SOD-525 and GPx-340 spectrophotometric assay kits according to the manufacturer’s instructions (Bioxytech; OXIS International, Portland, USA). Haemoglobin (Hb) concentrations were measured by a commercially available kit (Sigma, St. Louis, MO, USA).

### Statistical analyses

The data were analysed using the SPSS 15.0 program (SPSS, Chicago, IL). The significance of the differences between controls and patients end point means were analysed using Student’s *t* test. ANOVA (analysis of variance) was used for comparisons among the three or more groups. Multiple regression analysis was done to investigate the associations of the independent variables. Pearson correlation analysis was used for testing relationships between genotypes in patients and controls. The results were considered to be significant at *p* values of less than 0.05 (indicated by *). The differences in the distribution of genotype frequencies were calculated using the χ^2^ test. Genotype frequencies were checked for deviation from Hardy–Weinberg equilibrium and were not significantly different from those predicted. Odds ratios and 95 % confidence interval (95 % CI) were calculated to assess the relationship between *CYP1A1* and *GST* gene polymorphisms.

## Results

### General characteristic features of the study group

The general characteristic features of NSCLC patients (*n* = 246) who had no previous history of diagnosis and healthy controls (*n* = 250) included in this study are given in Table 1. NSCLC was predominant in males, affecting older men in the age group of 60–70 years. Age of onset of the disease was lower in women compared to males (55 vs. 58.91), although it was not statistically significant. The risk estimation for patients without co-morbid conditions compared to controls without co-morbid conditions was 3.58-fold (OR 3.58; 95 % CI 2.058, 6.24; *p* ≤ 0.001).

**Table 1:** General characteristics of the study group

Variables	Patients *n* = 246 (%)	Controls *n* = 250 (%)	*p* value
Gender			
Male	177 (71.95)	180 (73.91)	0.99
Female	69 (28.05)	70 (26.09)	0.99
Age			
Mean ± (SD)	57.57 ± 10.19	58.06 ± 9.56	
Stages of NSCLC			
II	28 (7.66)		
III	82 (34.23)		
IV	136 (58.11)		
Histology			
Squamous**-**cell carcinoma	97 (43.24)		
Adenocarcinoma	109 (48.65)		
Large cell and others	40 (8.11)		
Alcoholism			
Consumers	95 (38.62)*	26 (10.4)	0.01
Non-consumers	151 (61.38)*	224 (89.6)	0.01
Smoking status			
Never-smokers	98 (39.84)*	175 (69.08)	<0.001
Ex-smokers	42 (17.07)*	12 (4.35)	<0.001
Current smokers	106 (43.08) *	63 (26.57)	<0.001
≤20 pack years	38 (15.45)	28 (11.2)	0.16
21–40 pack years	34 (13.82)*	18 (7.2)	0.01
41–60 pack years	8 (3.25)	5 (18.85)	0.38
>60 pack years	26 (10.57)*	12 (7.72)	0.01
Passive smokers	38 (15.45)*	5 (2)	<0.001

* *p* < 0.05

### Molecular analysis of *CYP1A1 m1, m2*, *GSTM1* and *GSTT1* genes

**Fig. 1:**
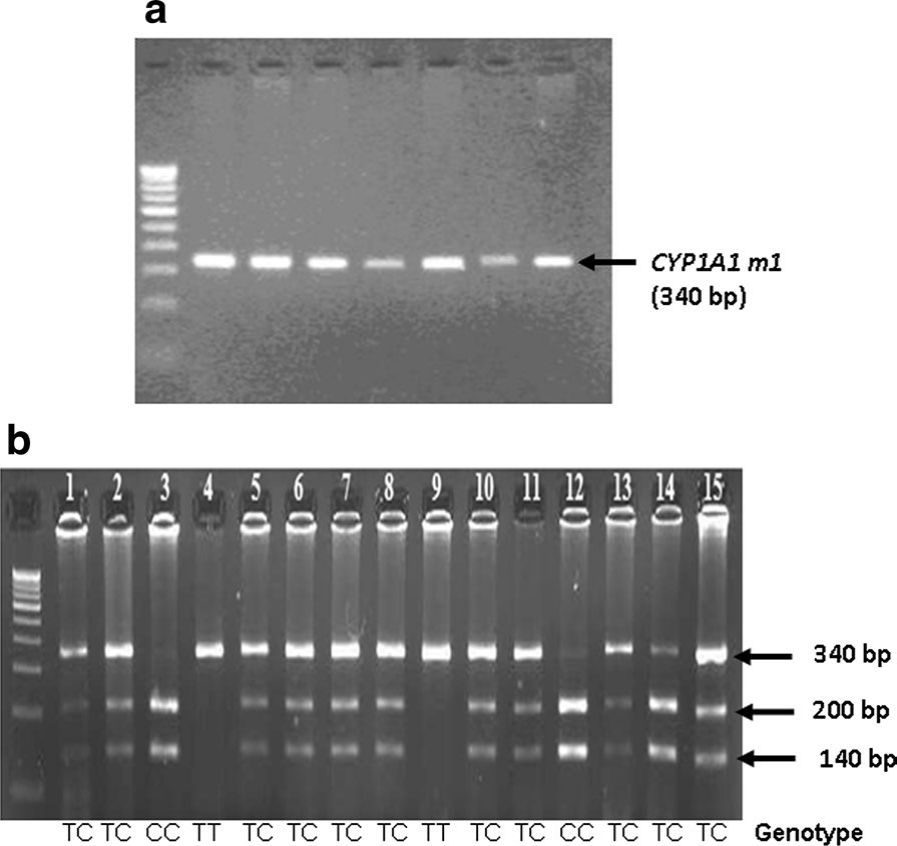
Amplifications of *CYP1A1 m1* and the RFLP products of the polymorphic forms: **a** PCR for *CYP1A1 m1* (340 bp) in multiple samples. **b** *CYP1A1 m1* polymorphisms were detected by RFLP. The 340-bp PCR product was digested with *Msp1* enzyme. *Lanes* *4* and *9* represent homozygous major type (TT; 340 bp); *Lanes* *3* and *12* represent homozygous minor (CC; 200 and 140 bp); *Lanes* *1*, *2*, *5*, *6*, *7*, *8*, *10*, *11*, *13*, *14* and *15* represent heterozygous type (TC; 340 bp, 200 and 140 bp)

**Fig. 2:**
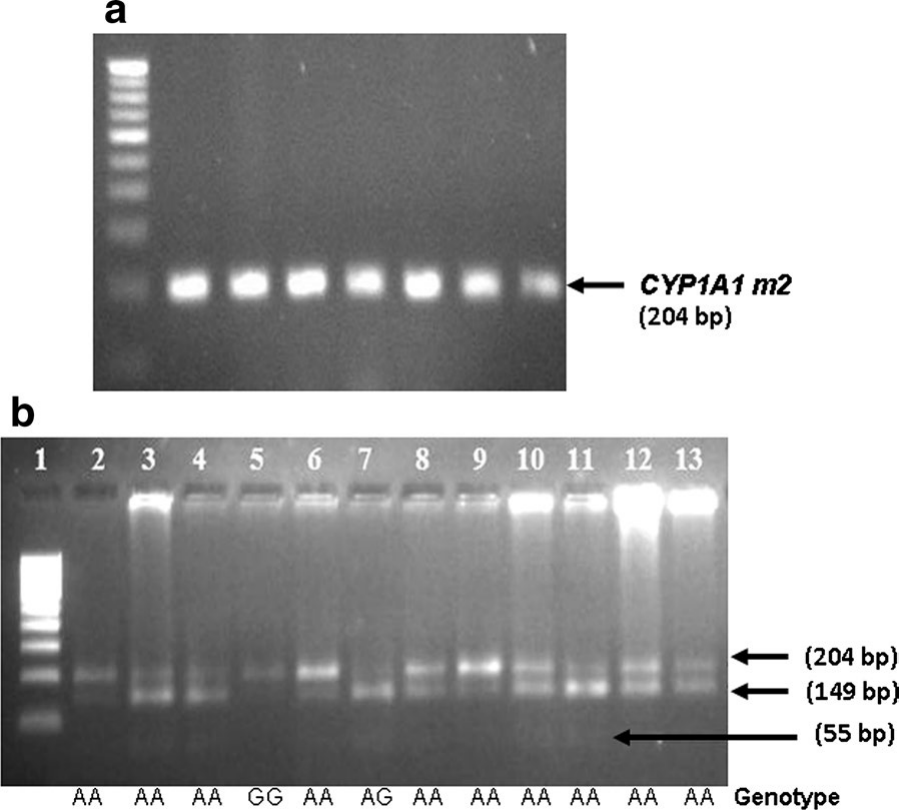
Amplifications of *CYP1A1 m2* and the RFLP products of the polymorphic forms: **a** PCR amplification for *CYP1A1 m2* (204 bp). **b** *CYP1A1 m2* polymorphism detected by RFLP. The 204-bp PCR product was digested with *BsrDI* enzyme. The cleavage site is lost in case of variants to give a single amplicon, whereas the wild-type allele generates 149- and 55-bp fragments. *Lane* *5* represents homozygous minor (GG); *Lane* *7* represents the heterozygote (AG); *Lanes* *2*–*4, 6* and *8*–*13* represent homozygous major (AA)

**Fig. 3:**
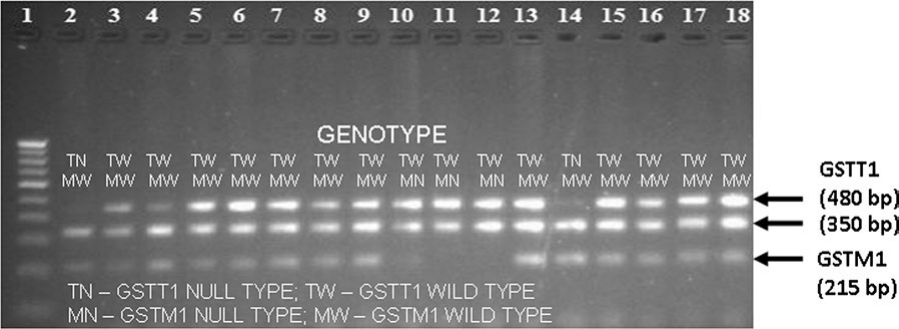
*GSTM1* and *GSTT1* polymorphisms: The *GSTM1* and *GSTT1* gene deletions were analysed simultaneously by multiplex PCR. Amplicons of 215 bp and 480 bp indicate, respectively, *GSTM1* and *GSTT1*. *Lane* *1*, 100-bp DNA ladder; *Lanes 2* and *14*, *GSTT1* Null type (TN); *Lanes 3*–*13* and *15–18*: *GSTT1* Wild type (TW); *Lanes 2–9* and *13–18:* *GSTM1* wild type (MW); *Lanes 10–12*: *GSTM1* Null type (MN). Albumin (350 bp) was used as an internal control

*CYP1A1m1* and m2 polymorphisms were detected by RFLP. PCR amplification for CYP1A1 *m1* produces 340-bp amplicons. A gain of *Msp1* restriction site in the polymorphic allele resulted in 340-bp products for homozygous major type (TT), 200 and 140 bp for homozygous minor (CC), respectively (Fig. 1). BsrD1 restriction enzyme-based digestion was used to detect the CYP1A1m2 polymorphisms. In the case of ‘GG’ (homozygous minor), due to loss of the restriction sites, a single amplicon of 204 bp was obtained, whereas in the ‘AA’ (homozygous major) allele will generate two amplicons of sizes, 149 and 55 bp (Fig. 2). Multiplex PCR-based approach was employed to determine the genetic polymorphisms of *GSTM1* and *GSTT1* genes. Amplicons of 215 bp and 480 bp indicated the presence of *GSTM1* and *GSTT1* genes (Fig. 3).

### Genotyping distribution of *CYP1A1 m1* (T3801C 3′ noncoding region)

The homozygous major (TT), heterozygous (TC) and homozygous minor (CC) genotype frequencies of *CYP1A1 m1* gene in healthy controls were 57.2, 37.2 and 5.6 %, respectively, whereas the same in NSCLC patients were 49.59, 38.62 and 11.78 %, respectively (Table 2). The ‘CC’ genotype was significantly higher in the NSCLC patients compared to healthy controls (*p* = 0.007, χ^2^ = 5.98, OR 2.25, 95 % CI 1.16–4.37) with 2.25-fold risk of disease susceptibility.

**Table 2:** Genotype and allelic distributions of the *CYP1A1 m1* gene polymorphisms in NSCLC patients and healthy controls

Genotype/Allele	Lung cancer (*n* = 246)	Controls (*n* = 250)	χ^2^	OR (95 % CI)	*p* value
	*N* (%)	*N* (%)			
TT	122 (49.59)	143 (57.2)	1	Reference	
TC	95 (38.62)	93 (37.2)	0.10	1.06 (0.73, 1.55)	0.42
CC	29 (11.78)	14 (5.6)	5.98	*2.25* (1.16, 4.37)	0.007*

* *p* < 0.05

### Genotyping distribution of *CYP1A1 m2* (Exon 7 Ile462Val)

The frequencies of *CYP1A1 m2* homozygous major (AA), heterozygous (AG) and homozygous minor (GG) genotypes in healthy controls were 78.4, 13.6 and 8 %, whereas the same were 29.67, 58.14 and 12.19 % in NSCLC patients, respectively (Table 3). Interestingly, the heterozygous ‘AG’ genotype was significantly higher in NSCLC group compared to healthy controls (*p* < 0.001, χ^2^ = 106.9, OR 8.82, 95 % CI 5.67–13.72) with an estimated 8.8-fold risk of developing lung cancer in individuals with this genotype.

**Table 3:** Genotype and allelic distribution of the *CYP1A1 m2* gene polymorphisms in NSCLC patients and healthy controls

Genotype/Allele	Lung cancer (*n* = 246)	Controls (*n* = 250)	χ^2^	OR (95 % CI)	*p* value
	*N* (%)	*N* (%)			
AA	73 (29.67)	196 (78.4)	1	Reference	
AG	143 (58.14)	34 (13.6)	106.9	*8.82* (5.67, 13.72)	0.001*
GG	30 (12.19)	20 (8)	2.43	1.59 (0.88, 2.89)	0.12

* *p* < 0.05

### Risk associated with additive effect of *CYP1A1 m1* and *CYP1A1 m2* polymorphisms within the same gene

Healthy controls displayed higher percentage of homozygous major (TT/AA) genotype combination (44.8 %) followed by the combination of homo/hetero (TT/AG; 38 %), and hetero/homo (TC/AA; 29.2 %), among all SNP combinations (Additional file 1: Table S1). Interestingly, in NSCLC patients, the frequency of homo/hetero genotypes and hetero/homo (TT/AG; 29.26 % and TC/AA; 14.63 %) was more common, followed by homozygous major (TT/AA; 13.41 %) genotypes. The frequency of homozygous minor genotype ‘CC/GG’ (*p* = 0.02, χ^2^ = 4.79, OR 12.48, CI 0.69–224.5) in patients demonstrated a 12-fold risk of developing lung cancer compared to the controls. The combination of ‘CC/AG’ (*p* = 0.004, χ^2^ = 6.89, OR 6.89, CI 2.01–23.6) showed a 6.9-fold risk of susceptibility to lung cancer, while heterozygous m1/m2 ‘TC/AG’ (*p* = 0.001, χ^2^ = 28.33, OR 5.18, CI 2.69–10.00) presented with a five fold risk.

### Genotypic distributions of *GSTM1* and *GSTT1* genes

*GSTM1* gene was found to be present in 76 % of the healthy controls and 73.98 % of NSCLC patients (Table 4). *GSTT1* gene was present in 89.6 % of healthy controls and 81.3 % of NSCLC patients and individuals lacking this gene were at a twofold risk of developing lung cancer (*p* = 0.008, χ^2^ = 6.86, OR 1.98, CI 1.18–3.32) (Table 5).

**Table 4:** Genotype distribution of the *GSTM1* gene polymorphism in NSCLC patients and healthy controls

Genotype	Lung cancer (*n* = 246)	Controls (*n* = 250)	χ^2^	OR (95 % CI)	*p* value
	*N* (%)	*N* (%)			
GSTM1 (+/+)	182 (73.98)	187 (76.0)	1	Reference	
GSTM1 (−/−)	64 (26.1)	63 (24.0)	0.04	1.04 (0.69, 1.56)	0.83

* *p* < 0.05

**Table 5:** Genotype distribution of the *GSTT1* gene polymorphism in NSCLC patients and healthy controls

Genotype	Lung cancer (*n* = 226)	Controls (*n* = 210)	χ^2^	OR (95 % CI)	*p* value
	*N* (%)	*N* (%)			
*GSTT1* (+/+)	200 (81.30)	224 (89.6)	1	Reference	
*GSTT1* (−/−)	46 (18.69)	26 (10.4)	6.86	*1.98* (1.18, 3.32)	0.008*

* *p* *<* 0.05

### Risk associated with combination of the two glutathione-S-transferase gene polymorphisms

Combined frequencies of *GSTM1* and *GSTT1* polymorphisms Wild/Wild, Wild/Null, Null/Wild and Null/Null in healthy control were 66.80, 10.40, 23.60 and 1.6 %, respectively, whereas in NSCLC patients the frequencies were 59.34, 14.63, 21.95 and 4.06 %, respectively. It was clear that the GSTM1 Wild/GSTT1 Wild genotype followed by GSTM1 Null/GSTT1 Wild combinations were more predominant in both healthy controls and NSCLC patients (Additional file 1: Table S2). The disease association was found between GSTM1 Wild/GSTT1 Null genotype (*p* = 0.01, OR 1.97) and GSTM1 Null/GSTT1 Null (*p* = 0.04, OR 2.6) combinations, indicating a 1.97- and 2.6-fold risk of disease susceptibility, respectively (Additional file 1: Table S2).

### Risk of NSCLC associated with *CYP1A1*, *GSTM1* and *GSTT1* genotypes stratified by smoking exposure

Patients who were non-smokers and having a *CYP1A1 m1* (T/C) (OR 1.82, 95 % CI 1.08, 3.07) and *CYP1A1 m2* (A/G) (OR 12.39 95 % CI 6.53, 23.51) genotypes had an increased lung cancer. Lung cancer patients who smoked and having *CYP1A1 m1* T/C, C/C and *CYP1A1 m2* A/G, G/G and *GSTT1* null (−/−) genotypes were at higher risk compared to the controls (Table 6).

**Table 6:** Risk of NSCLC associated with *CYP1A1 m1*, m2, *GSTM1*, *GSTT1* genotypes stratified by smoking exposure

Variables smoking status	Cases/ controls non-smokers 98/175	OR (95 % CI) non-smokers	*p*	Cases/ controls smokers 148/75	OR (95 % CI) smokers	*p* value
*CYP1A1m1* (T/T)	49/115	0.52 (0.31, 0.86)	0.01	63/58	0.21 (0.11, 0.40)	<0.001
*CYP1A1m1* (T/C)	40/48	*1.82* (1.08, 3.07)	0.02*	70/15	*3.59* (1.87, 6.88)	<*0.001* *
*CYP1A1m1* (C/C)	9/12	1.37 (0.56, 3.38)	0.48	15/2	*4.11* (0.91,18.5)	0.04*^,#^
*CYP1A1m2* (A/A)	33/142	0.11 (0.06, 0.20)	0.001	40/54	0.14 (0.07, 0.26)	<0.001
*CYP1A1m2* (A/G)	56/17	*12.39* (6.53, 23.51)	0.001*	87/17	*4.86* (2.58, 9.15)	<0.001*
*CYP1A1m2* (G/G)	9/16	1.05 (0.42, 2.36)	0.99	21/4	*2.93* (0.96, 8.88)	0.04*^,#^
*GSTM1* (+/+)	76/133	1.09 (0.60, 1.96)	0.77	106/54	0.98 (0.52, 1.82)	0.95
*GSTM1* (−/−)	22/42	0.92 (0.51, 1.65)	0.77	42/21	1.01 (0.54, 1.89)	0.95
*GSTT1* (+/+)	89/158	1.06 (0.45, 2.48)	0.88	111/66	0.40 (0.18, 0.90)	0.02
*GSTT1* (-/-)	9/17	0.93 (0.40, 2.19)	0.88	37/9	*2.44* (1.11, 5.38)	0.02*

* *p* < 0.05

^#^Yates corrected Chi square

### Association of *CYP1A1*, *GSTM1* and *GSTT1* genotypes stratified by histology

In all the three pathological subtypes, *CYP1A1m2* A/G, *GSTM1* (+/+) wild and *GSTT1* (+/+) wild were the predominant genotypes (Table 7).

**Table 7:** Genotypic distributions of *CYP1A1 m1, m2*, *GSTM1*, *GSTT1* stratified by histology of NSCLC

Genotype	Squamous	%	Adeno	%	Large + others	%
*CYP1A1m1* (T/T)	45	46.4	48	44	12	30
*CYP1A1m1* (T/C)	46	47.4	52	47.7	21	52.5
*CYP1A1m1* (C/C)	6	6.2	9	8.3	7	17.5
*CYP1A1m2* (A/A)	25	25.8	37	33.9	11	27.5
*CYP1A1m2* (A/G)	62	63.9	56	51.4	25	62.5
*CYP1A1m2* (G/G)	10	10.3	16	14.7	4	10
*GSTM1* (+/+)	69	71.1	80	73.4	33	82.5
*GSTM1* (−/−)	28	28.9	29	26.6	7	17.5
*GSTT1* (+/+)	78	80.4	88	80.7	34	85
*GSTT1* (−/−)	19	19.6	21	19.3	6	15

### Risk of NSCLC associations with combination of *CYP1A1* and *GST* genes

The combinations of genotypes having a profound effect were *CYP1A1 m2* A/G + *GSTM1* wild (+/+); *CYP1A1 m2* A/G + *GSTM1* null (−/−); and *CYP1A1 m2* G/G + *GSTT1* wild (+/+) with estimated risks of sixfold, sixfold and 10.5-fold, respectively (Additional file 1: Table S3).

In the case of three genotype combinations, *CYP1A1 m1* T/T + *CYP1A1 m2* G/G + *GSTM1* null (−/−) showed a 19-fold risk; *CYP1A1 m1* T/C + *CYP1A1 m2* G/G + *GSTM1* wild (+/+) showed 11.6-fold risk and *CYP1A1 m1* G/G + *CYP1A1 m2* A/G + *GSTM1* wild (+/+) showed a 10.5-fold risk of disease susceptibility (Additional file 1: Table S4).

The overall risk of NSCLC associated with three genotype combinations of *CYP1A1 m1, m2* and/or *GSTT1* genes ranged from 3.48 to 10.55 (Additional file 1: Tables S5, S6). When analysed for the overall risk with four genotype combinations, it ranged from 5.22 to 13.89 (Additional file 1: Tables S7–S9). Spearman coefficient correlation indicated *CYP1A1 m2* gene significantly correlated with *GSTM1* and GSTT1 genes (Table 8).

**Table 8:** Spearman correlation coefficients between genotypes in NSCLC patients and healthy controls

	*CYP1A1m1*	*CYP1A1m2*	*GSTM1*	*GSTT1*
*CYP1A1m1*	1	−0.005	−0.123	−0.074
	–	0.935	0.054	0.250
*CYP1A1m2*	−0.005	1	0.152^a^	−0.167^b^
	0.935	–	0.017	0.009
*GSTM1*	−0.123	0.152^a^	1	−0.047
	0.054	0.017	–	0.465
*GSTT1*	−0.074	−0.167^b^	−0.047	1
	0.250	0.009	0.465	–

^a^Correlation is significant at the 0.05 level (2-tailed)

^b^Correlation is significant at the 0.01 level (2-tailed)

### Impact of gene polymorphisms on oxidative stress markers

The impact of *CYP1A1 m1*, *CYP1A1m2*, *GSTM1* and *GSTT1* gene polymorphisms on superoxide dismutase activity (Table 9), Glutathione peroxidase activity (Table 10), MDA (Table 11) and 8-OHdG levels (Table 12) were assessed between controls and lung cancer patients. In NSCLC patients, there was a significant difference between the SOD levels of *GSTT1* wild (+/+) vs null (−/−); GPx activities between *CYP1A1 m1* T/T vs T/C and T/T vs C/C; *CYP1A1 m2* A/A vs A/G and A/A vs G/G genotypes and *GSTT1* wild vs null genotypes. Mean MDA levels were significantly different with respect to *CYP1A1 m1* and *GSTM1* genotypes. The difference in 8-OHdG levels between the genotypes was significant only for *CYP1A1 m*2 gene and *GSTT1* gene polymorphisms. The difference between the genotypes of different genes for SOD, GPx, MDA and 8-OHdG levels were not significant in the control group.

**Table 9:** Association of superoxide dismutase levels in relation to *CYP1A1* and *GST* gene polymorphisms in NSCLC patients and healthy controls

Gene	Genotype	Lung cancer patients (SOD levels)		*p* value	Controls (SOD levels)		*p* value	Total *p* value
		*n*	Mean ± SE		*n*	Mean ± SE		
*CYP1A1*, *m*1	T/T	121	914.28 ± 30.05		138	1166.08 ± 12.8		0.92
	T/C	89	915.73 ± 17.03	0.45	98	1165.71 ± 13.93	0.35	0.14
	C/C	28	910.74 ± 13.57	0.62	14	1070.00 ± 37.60	0.78	0.65
*CYP1A1*, *m2*	A/A	71	922.25 ± 18.18		196	1190.00 ± 10.61		<0.01*
	A/G	156	909.87 ± 12.00	0.81	34	1165.91 ± 20.61	0.14	0.05*
	G/G	11	898.18 ± 67.01	0.07	20	1080.58 ± 23.82	0.08	0.02*
*GSTM1*	+/+	174	927.01 ± 11.81		187	1214.54 ± 7.47		<0.01*
	–/–	64	875.00 ± 18.00	0.56	63	1175.71 ± 10.72	0.15	<0.01*
*GSTT1*	+/+	192	929.58 ± 10.57		224	1168.03 ± 9.86		0.91
	–/–	46	843.91 ± 24.69	0.04*	26	1096.12 ± 23.16	0.10	0.05*

* *p* < 0.05

**Table 10:** Association of glutathione peroxidase activity in relation to *CYP1A1* and *GST* gene polymorphisms in NSCLC patients and healthy controls

Gene	Genotype	Lung cancer patients (GPx levels)		*p* value	Controls (GPx levels)		*p* value	Total *p* value
		*n*	Mean ± SE		*n*	Mean ± SE		
*CYP1A1* *m*1	T/T	121	47.51 ± 4.52		138	55.44 ± 0.60		<0.01*
	T/C	89	45.77 ± 2.45	0.01*	98	55.15 ± 0.69	0.74	<0.01*
	C/C	28	43.42 ± 2.02	<0.01*	14	51.42 ± 1.74	0.79	0.06
*CYP1A1* *m2*	A/A	71	48.18 ± 6.45		196	59.35 ± 0.33		<0.01*
	A/G	156	44.02 ± 1.75	<0.01*	34	55.26 ± 0.65	0.17	<0.02*
	G/G	11	45.94 ± 2.93	0.01*	20	54.67 ± 0.91	0.53	<0.01*
*GSTM1*	+/+	174	46.22 ± 1.74		187	57.69 ± 0.36		<0.01*
	–/–	64	40.87 ± 2.69	0.33	63	54.41 ± 0.84	0.21	<0.01*
*GSTT1*	+/+	192	46.66 ± 1.53		224	55.50 ± 0.47		<0.001*
	–/–	46	36.95 ± 3.95	0.05*	26	51.69 ± 1.12	0.07	<0.001*

* *p* < 0.05

**Table 11:** Association of Lipid peroxidation (MDA) levels in relation to *CYP1A1* and *GST* gene polymorphisms in NSCLC patients and healthy controls

Gene	Genotype	Lung cancer patients (MDA levels)		*p* value	Controls (MDA levels)		*p* value	Total *p* value
		*n*	Mean ± SE		*n*	Mean ± SE		
*CYP1A1* *m*1	T/T	127	4.18 ± 0.24		138	1.72 ± 0.10		0.01*
	T/C	90	4.21 ± 0.13	<0.01*	98	1.79 ± 0.24	0.42	<0.01*
	C/C	29	4.30 ± 0.13	<0.01*	14	2.00 ± 0.42	0.83	<0.01*
*CYP1A1* *m2*	A/A	73	4.23 ± 0.15		196	1.56 ± 0.11		*0.07*
	A/G	162	4.22 ± 0.11	0.39	34	1.75 ± 0.23	0.34	0.79
	G/G	11	4.52 ± 0.39	0.87	20	2.12 ± 0.28	0.29	0.86
*GSTM1*	+/+	182	4.11 ± 0.10		187	1.73 ± 0.03		<0.01*
	–/–	64	4.60 ± 0.13	0.02*	63	1.95 ± 0.71	0.21	<0.001*
*GSTT1*	+/+	200	4.06 ± 0.09		224	1.75 ± 0.29		<0.001*
	–/–	46	5.00 ± 0.20	0.31	26	1.91 ± 0.12	0.09	<0.01*

* *p* < 0.05

**Table 12:** Urinary 8-OHdG levels (ng/mg creatinine) in relation to *CYP1A1* and *GST* gene polymorphisms in NSCLC patients and healthy controls

Gene	Genotype	Lung cancer patients (8-OhdG levels)		*p* value	Controls (8-OhdG levels)		*p* value	Total *p* value
		*n*	Mean ± SE		*n*	Mean ± SE		
*CYP1A1* *m*1	T/T	64	6.03 ± 0.09		57	4.22 ± 0.10		0.71
	T/C	45	6.08 ± 0.12	0.41	37	4.44 ± 0.11	0.44	0.25
	C/C	16	6.11 ± 0.23	0.18	6	4.50 ± 0.19	0.27	0.13
*CYP1A1* *m2*	A/A	41	5.91 ± 0.21		79	4.28 ± 0.17		0.42
	A/G	78	6.03 ± 0.09	0.007*	17	4.37 ± 0.17	0.47	0.58
	G/G	6	6.13 ± 0.13	0.04*	4	4.75 ± 0.19	0.45	0.67^#^
*GSTM1*	+/+	102	5.97 ± 0.78		71	4.26 ± 0.85		0.39
	–/–	23	6.43 ± 0.16	0.60	29	4.61 ± 0.10	0.28	0.07
*GSTT1*	+/+	101	5.88 ± 0.69		88	4.23 ± 0.64		0.16
	–/–	24	6.82 ± 0.17	0.02*	12	5.35 ± 0.10	0.63	0.04*

* *p* < 0.05

^#^yates corrected Chi square

### Multiple regression analysis of different variants in lung cancer patients

Multiple regression analysis was performed by taking age, gender, smoking status, alcohol consumption, dietary habits, occupation, family history, stage of the disease and histology (Table 13). We observed that smoking, histology, stage of the disease, MDA levels, GPx activities and polymorphisms in *CYP1A1 m1* and *GSTT1* genes were the strongest predicting factors for increased free radical generation and imbalances in antioxidant defence causing oxidative stress and leading to disease susceptibility in lung cancer patients. Other variables did not have any impact as reflected by lack of significance.

**Table 13:** Multiple regression analysis of different variants in NSCLC

Variable	Unstandardised coeeficients		Standardized coefficients	*t*	Sig.	95 % confidence interval for *B*	
						Lower bound	Upper bound
	*B* ^a^	Standard error^b^					
Age	0.001	0.001	0.012	1.241	0.216	0.000	0.002
Sex	−0.017	0.014	−0.015	−1.245	0.215	−0.044	0.010
Smoking	−0.005	0.008	−0.009	−0.624	0.034*	−0.021	0.011
Passive smoking	−0.003	0.018	−0.002	−0.165	0.869	−0.038	0.032
Histology	−0.049	0.009	−0.094	−5.402	0.000*	−0.067	−0.031
Stage	−0.173	0.017	−0.463	−9.935	0.000*	−0.207	−0.138
Alcohol	0.007	0.011	0.007	0.639	0.523	−0.015	0.030
Diet	−0.001	0.013	−0.001	−0.063	0.950	−0.026	0.025
Place of living	−0.004	0.006	−0.007	−0.666	0.506	−0.015	0.007
Familial	−0.018	0.023	−0.008	−0.769	0.443	−0.063	0.027
MDA	0.026	0.013	0.083	1.991	0.048*	0.000	0.052
GPx	−0.005	0.001	−0.199	−9.468	0.000*	−0.006	−0.004
SOD	−0.005	0.000	0.012	0.494	0.622	0.000	0.000
8-OHdG	0.019	0.012	0.045	1.622	0.106	−0.004	0.043
CYPM1	−0.003	0.007	−0.003	−0.376	0.007*	−0.016	0.011
CYPM2	−0.002	0.008	−0.003	−0.268	0.789	−0.019	0.014
*GSTM1*	−0.008	0.015	−0.006	−0.512	0.609	−0.037	0.022
*GSTT1*	−0.020	0.018	−0.014	−1.072	0.025*	−0.056	0.017

^a^Slope of the regression line

^b^Standard error of the regression line

* *p* < 0.05

## Discussion

Xenobiotic metabolising enzymes expedites purging of a variety of toxic substances, thereby gaining prominence in the pathophysiology of cancer. Hence, gene polymorphisms in the enzymes that are intricate in the metabolism of carcinogens may regulate an individual’s predisposition to cancer including lung cancer [36]. Besides this, environmental and life style insults also contribute to the predisposition of lung cancer. Cigarette smoke contain PAHs which can be metabolically activated to highly reactive compounds capable of binding to DNA and initiating the carcinogenic process [37, 38]. Among the variety of xenobiotic metabolising enzymes, *CYP1A1*, *GSTM1* and *GSTT1* have been implicated to modulate the risk of lung cancer because of their potential involvement in carcinogenesis metabolism. Globally many studies reported on the association among gene interactions and lung cancer in different populations, but the conclusions were conflicting [18]. In the Indian context, risk assessment between gene polymorphisms and lung cancer was investigated in Northern and Southern Indian populations. *CYP1A1*, *GSTM1* and *GSTT1* polymorphisms and the association with lung cancer in the South Indian population (patients reporting to a specific hospital in Thiruvananthapuram, the capital city of Kerala state) was reported [30], suggesting the risk in the specific population of that state. However, there are genotypic, life style and environmental differences in the populations of the five states (Andhra Pradesh, Tamilnadu, Kerala, Karnataka and Maharashtra) of South India. Hence, we conducted systematic analyses on the associations of *CYP1A1*, *GSTM1* and *GSTT1* polymorphisms with the risk of NSCLC in the population of Andhra Pradesh.

In the present study, a high frequency of *CYP1A1 m1* homozygous minor genotype (C/C) was recorded among NSCLC patients. Association of lung cancer risk with homozygosity of *CYP1A1* variant alleles was reported in Chilean and Caucasian populations [39–41]. Likewise, in the North and South Indian populations, the association of *CYP1A1* polymorphism with lung cancer risk was reported [30, 42, 43]. Further, in the current study, evaluation of the genotypic frequencies in lung cancer patients from Andhra Pradesh have shown a higher frequency and a significant association of *CYP1A1 m2* heterozygous ‘AG’ genotype. Similar observations (higher frequency of *CYP1A1 m2* (A/G) allele) were reported in lung cancer patients from Korea [19]. On the same lines, *CYP1A1 m2* (G/G) allele frequency was demonstrated to be lower in Caucasians than Japanese [44]. Heterozygous and homozygous minor *CYP1A1 m2* genotypes were on the higher side in Chilean lung cancer patients [25]. The higher frequency of this gene was also reported in the Southern and Northern Indian lung cancer patients [42, 45]. Results of our study are in parallel with the observations made in different populations worldwide, and it is possible that the mutated genotype of *CYP1A1* plays an important role in the aetiology of lung cancer in the population of Andhra Pradesh state.

In the current study, *GSTM1* wild and null genotypes were detected, respectively, in 73.98 and 26.1 % of lung cancer patients. A similar trend was observed in the healthy controls (76 % wild type and 24 % null type, respectively). Similarly, *GSTM1* null genotype was not associated with the increased risk of lung cancer, and the proportions of the NSCLC patients and healthy controls exhibiting *GSTM1* null genotype were apparently equal. Similar trends were observed in the South and North Indian population cohorts [30, 46]. On the contrary, *GSTM1* null or deletion genotype was reported to be prevalent in about 50 % of Caucasians, 33 % of African Americans and 45 % of Japanese [47] lung cancer patients. We found that *GSTT1* null genotype was high in lung cancer patients compared to the controls, which is consistent [30, 41, 48] and in conflict [42, 49] with previous reports. *GSTT1* deletion polymorphisms was reported in 13–28 % of Caucasians [18]. Similarly, the frequencies of homozygous deletions (null genotype) for *GSTM1* and *GSTT1* were found to be 22.4 % and 17.6 %, respectively, in the South Indian population; 54 % and 13 %, respectively, in the East Indian population [50, 51]; 41 % and 21.5 %, respectively, in the North Indian population [46]. Results of our study and others indicate that in the Indian context, the risk of lung cancer is more associated with *GSTT1* polymorphism rather than *GSTM1* genotype.

A majority of the patients included in our study were bidi smokers (made of crude particles of dried tobacco leaves wrapped in a tendu or temburni leaf and rich in tar and nicotines); and bidi smoking is known to generate stronger carcinogen load than cigarette [52]. Our data clearly indicate that individuals who were smokers and had *CYP1A1m1* T/C, C/C or *CYP1A1m2* A/G, G/G genotypes and GSTT1 null genes were at higher risk of disease susceptibility to lung cancer. A threefold risk of lung cancer associated with *CYP1A1 m1* genotype was reported [46]. Further, increased risk of lung cancer in heavy smokers [34, 42] and light smokers [20, 53] with *CYP1A1 m1* allele was demonstrated. In the present study, no risk of lung cancer was associated with *GSTM1* null genotype in smokers and non-smokers. The association between *GSTM1* genotype and cumulative smoking is controversial [47]. Stronger associations were reported in casual smokers [42] and low smoking exposed individuals [53], whereas such an association was not evident in other reports [54]. Results of our study indicate that *CYP1A1* polymorphisms rather than *GSTM1* polymorphisms and smoking contribute to the higher risk of lung cancer. Our results are in accordance with another study from North India where the relative risk for the carriers of variant *CYP1A1* genotypes was high [55]. The disease association among combination of GST genes in the lung cancer patients was found between the Wild/Null and Null/Null types. The increased risk due to deletions of GST may result in less detoxification of xenobiotics, thereby making the individual more susceptible to toxic substances present in the environment.

Analysing multiple gene interactions provide better understanding to assess the risks associated with lung cancer risk. In our study, the combinations of two (*CYP1A1 m2* and *GSTM1*) or three (*CYP1A1 m1, CYP1A1 m2* and *GSTM1/GSTT1*) genotypes had a profound effect on susceptibility to lung cancer up to 14-fold depending on the genotypic interaction. Correlations between lung cancer risk and combinations of *CYP1A1*, *GSTM1* and *GSTT1* is of particular interest since these genotypes suggest that alterations in the action of phases I and II enzymes lead to defective metabolism of xenobiotic compounds, thereby potentiating the cancer risk. It was suggested that individuals having polymorphisms in more than one of these genes are at higher risk than having for only one gene [56]. Polymorphisms of MspI and exon7-Val of *CYP1A1* and *GSTM1* null genotypes and increased lung cancer risk was evidenced in summarized data of 46 studies of Chinese populations [56]. In an Indian population study, a twofold risk of lung cancer was found in individuals displaying variations in the *CYP1A1* and *GSTM1* genes [42]. *CYP1A1*, *GSTM1*, GSTP1 and *GSTT1* polymorphisms and their association to lung cancer in a cohort of North Indian population was reported [46]. Similarly, in a study involving South Indian population, a 4.4-fold increased risk of the *GSTM1* null, *GSTT1* null, *CYP1A1* homozygous major genotype combination and a 3.5-fold increased risk, although not statistically significant, in individuals possessing the *GSTM1* null, *GSTT1* null, *CYP1A1* homozygous minor genotype combination [30, 57] were reported. Results of our study are in agreement with the reported data and clearly indicate strong associations of *CYP1A1*, *GSTM1* and *GSTT1* genetic polymorphism with NSCLC.

The role of oxidative stress in the pathophysiology of a variety of cancers including lung cancer was well documented [32]. Genetic polymorphisms of metabolic enzymes and oxidative stress markers in occupational exposure were reported [58]. However, the association between oxidative stress and genetic polymorphisms with respect to lung cancer was not documented. We previously demonstrated that 8-oxo-dG and malondialdehyde levels were increased and red cell superoxide dismutase and glutathione peroxidase activities were significantly decreased in lung cancer patients [32]. Hence, in this study, the genotypes of polymorphic markers were stratified with respect to oxidative markers to evaluate whether the inter-individual variation of oxidants and antioxidants could lead to disease susceptibility. To the best of our knowledge, this is the first study to assess the association of polymorphism of *CYP1A1* and *GST* genes with respect to SOD, GPx, MDA and 8-oxo-dG levels in lung cancer patients from India. We found an association between *GSTT1* null genotype and SOD activity, *CYP1A1 m1, m2* and *GSTT1* and GPx activity, MDA levels and *CYP1A1 m1* & *GSTM1*, 8-oxo-dG and *CYP1A1 m1* and *GSTT1* gene polymorphisms. Although no information is available on the association of oxidants, antioxidants and gene polymorphisms, some information is available on the association of gene polymorphisms and urinay 8-oxo-dG levels. While some studies [59–65] demonstrated the influence of gene polymorphisms on urinary 8-oxo-dG levels, some other studies did not show such an association [66–68]. It is possible that deletion polymorphisms of *GSTM1* and *GSTT1* (null genotype) results in no functional enzymatic activity, thereby failing to detoxify several xenobiotics including tobacco smoke constituents and finally leading to increased generation of ROS and lowered GPx activity and ${\text{O}}_{2}^{ - }$ scavenging activity of SOD. Results of our study provide strong association between gene polymorphisms, oxidant and antioxidant status and the risk of developing NSCLC, which hitherto was not reported.

The limitations of our study are that the healthy controls and NSCLC patients were in the ratio of 1:1, and some of the NSCLC patients had co-morbid conditions. Further studies with large sample size can provide concrete data on the combined effect of genetic polymorphisms and NSCLC. The effect of co-morbid conditions to the contribution of gene polymorphisms observed cannot be ruled out.

In conclusion, we report that in the population of Andhra Pradesh, the South Indian state, a higher risk of lung cancer was associated with combined gene polymorphisms of phase I and phase II enzymes, than with a single susceptible gene. Risk assessment of NSCLC can be related to gene polymorphisms and oxidant status. This finding may have an important implication for the prevention of smoking and occupational exposures in susceptible individuals.

## Additional file

10.1186/s40001-016-0209-x Risk associated with the combination of gene polymorphisms in *CYP1A1 m1*, *m2*, *GSTM1* and *GSTT1* genes.
