# Supplementary material for: Association of CYP1A1, GSTM1 and GSTT1 gene polymorphisms with risk of non-small cell lung cancer in Andhra Pradesh region of South India
Source: Eur J Med Res. 2016 Apr 18;21:17. doi: 10.1186/s40001-016-0209-x (PMC4836067; doi:10.1186/s40001-016-0209-x)
Supplement: Supplementary file 1 — 10.1186/s40001-016-0209-x Risk associated with the combination of gene polymorphisms in CYP1A1 m1, m2, GSTM1 and GSTT1 genes. [file 40001_2016_209_MOESM1_ESM.docx]

**Table S1: Distribution of *CYP1A1 m1* and *m2* gene polymorphisms in NSCLC patients**

| **Type of combinations**  CYP1A1m1/ CYP1A1m2 | **Lung Cancer (N=246)**    **N %** | **Control (N=250)**  **N %** | **χ^2^** | **OR (95% CI)** | ***P-value*** |
| --- | --- | --- | --- | --- | --- |
| TT/AA | 33 13.41 | 112 44.8 | 58.92 | 0.19 (0.12, 0.29) | <0.001 |
| TT/AG | 72 29.26 | 19 38 | 38.78 | **5.03 (2.92, 8.65)** | **<0.001*** |
| TT/GG | 16 6.50 | 12 4.8 | 0.67 | 1.38 (0.63, 2.98) | 0.41 |
| TC/AA | 36 14.63 | 73 29.2 | 15.31 | 0.41 (0.26, 0.64) | <0.001 |
| TC/AG | 51 20.73 | 12 4.8 | 28.33 | **5.18** (2.69, 10.00) | **<0.001*** |
| TC/GG | 8 3.25 | 8 3.1 | 0.01 | 1.01 (0.37, 2.75) | 0.97 |
| CC/AA | 5 2.03 | 11 4.4 | 2.22 | 0.45 (0.15, 1.31) | 0.13 |
| CC/AG | 19 7.72 | 3 1.2 | 12.42 | **6.89** (2.01, 23.6) | **0.004*^#^** |
| CC/GG | 6 2.44 | 0 0 | - | **-** | **-** |

***p<0.05; ^#^ yates corrected chi square**

**Table S2: Distribution of combination of *GSTM1* and *GSTT1* genes**

**in NSCLC patients and healthy controls**

| **Type of combination**  **GSTM1/GSTT1** | **Lung cancer**  **(N= 246)**  **N %** | **Control (N=250)**  **N %** | **χ2** | **OR (95% CI)** | ***P-value*** |
| --- | --- | --- | --- | --- | --- |
| **Wild (++) / Wild (++)** | 146 59.34 | 143 54.8 | 0.23 | 1.09 (0.76 - 1.56) | 0.62 |
| **Wild (++) / Null (--)** | 36 14.63 | 20 10.4 | 5.43 | **1.97** (1.10 – 3.51) | **0.01*** |
| **Null (--) / Wild (++)** | 54 21.95 | 59 23.6 | 0.19 | 0.91 (0.59 - 1.38) | 0.66 |
| **Null (--) / Null* (--)** | 10 4.06 | 4 1.6 | 2.74 | **2.6** (0.80 – 8.42) | **0.04*^#^** |

***p<0.05; ^#^ yates corrected chi square**

**Table S3: Risk of NSCLC associated with two genotype combinations (*CYP1A1* and *GST*)**

| **Genotype combination** | **Cases/ Controls** | **OR (95% CI)** | **p** |
| --- | --- | --- | --- |
| ***CYP1A1 m1* (T/T) + *GSTM1* (+/+) wild** | 74/133 | 0.37 (0.26, 0.54) | <0.001 |
| *CYP1A1 m1* (T/T) + *GSTM1* (-/-) null | 31/40 | 0.75 (0.45, 1.25) | 0.34 |
| *CYP1A1 m1* (T/C) + *GSTM1* (+/+) wild | 84/44 | **2.42** (1.59, 3.69) | **<0.001*** |
| *CYP1A1 m1* (T/C) + *GSTM1* (-/-) null | 32/19 | **1.81** (0.96, 3.5) | **0.04*** |
| *CYP1A1 m1* (C/C) + *GSTM1* (+/+) wild | 21/12 | 1.85 (0.89, 3.85) | 0.09 |
| *CYP1A1 m1* (C/C) + *GSTM1* (-/-) null | 4/2 | 2.05 (0.37, 11.29) | 0.40^#^ |
| ***CYP1A1 m1* (T/T) + *GSTT1* (+/+) wild** | 84/155 | 0.31 (0.22, 0.45) | <0.001 |
| *CYP1A1 m1* (T/T) + *GSTT1* (-/-) null | 21/18 | 1.20 (0.62, 2.31) | 0.58 |
| *CYP1A1 m1* (T/C) + *GSTT1* (+/+) wild | 94/58 | **2.04** (1.38, 3.02) | **<0.001*** |
| *CYP1A1 m1* (T/C) + *GSTT1* (-/-) null | 24/6 | **4.39** (1.76, 10.95) | **<0.001*** |
| *CYP1A1 m1* (C/C) + *GSTT1* (+/+) wild | 21/12 | 1.85 (0.89, 3.85) | 0.09 |
| *CYP1A1 m1* (C/C) + *GSTT1* (-/-) null | 2/1 | 2.04 (0.18, 22.65) | 0.55^#^ |
| ***CYP1A1 m2* (A/A) + *GSTM1* (+/+) wild** | 63/142 | 0.26 (0.17, 0.38) | <0.001 |
| *CYP1A1 m2* (A/A) + *GSTM1* (-/-) null | 10/54 | 0.15 (0.075, 0.31) | <0.001 |
| *CYP1A1 m2* (A/G) + *GSTM1* (+/+) wild | 98/25 | **5.95** (3.66, 9.68) | **<0.001*** |
| *CYP1A1 m2* (A/G) + *GSTM1* (-/-) null | 45/9 | **5.99** (2.86, 12.56) | **<0.001*** |
| *CYP1A1 m2* (G/G) + *GSTM1* (+/+) wild | 21/20 | 1.07 (0.56, 2.03) | 0.82 |
| *CYP1A1 m2* (G/G) + *GSTM1* (-/-) null | 9/0 | **-** | **-** |
| ***CYP1A1 m2* (A/A) + *GSTT1* (+/+) wild** | 49/180 | 0.09 (0.06, 0.14) | <0.001 |
| *CYP1A1 m2* (A/A) + *GSTT1* (-/-) null | 24/16 | 1.58 (0.45, 1.25) | 0.17 |
| *CYP1A1 m2* (A/G) + *GSTT1* (+/+) wild | 127/24 | **10.5** (0.45, 1.25) | **<0.001*** |
| *CYP1A1 m2* (A/G) + *GSTT1* (-/-) null | 16/10 | 1.67 (0.45, 1.25) | 0.21 |
| *CYP1A1 m2* (G/G) + *GSTT1* (+/+) wild | 24/20 | 1.24 (0.45, 1.25) | 0.49 |
| *CYP1A1 m2* (G/G) + *GSTT1* (-/-) null | 6/0 | **-** | **-** |

***p<0.05; ^#^ yates corrected chi square**

**Table S4: Risk of NSCLC associated with combination of three genotypes (*CYP1A1m1*, *CYP1A1m2* and *GSTM1*)**

| **Genotype combination** | **Cases/ Controls** | **OR (95% CI)** | **p** |
| --- | --- | --- | --- |
| *CYP1A1 m1* T/T + *CYP1A1 m2* A/A + *GSTM1* wild (+/+) | 22/97 | 0.15 (0.09, 0.25) | <0.001 |
| *CYP1A1 m1* T/T + *CYP1A1 m2* A/A + *GSTM1* null (-/-) | 4/37 | 0.09 (0.03, 0.27) | 0.01**^#^** |
| *CYP1A1 m1* T/T + *CYP1A1 m2* A/G + *GSTM1* wild (+/+) | 46/17 | **3.15** (1.75, 5.67) | **<0.01*** |
| *CYP1A1 m1* T/T + *CYP1A1 m2* A/G + *GSTM1* null (-/-) | 18/3 | **6.5** (1.89, 22.36) | **<0.001***^#^ |
| *CYP1A1 m1* T/T + *CYP1A1 m2* G/G + *GSTM1* wild (+/+) | 5/19 | 0.25 (0.09, 0.68) | 0.003 |
| *CYP1A1 m1* T/T + *CYP1A1 m2* G/G + *GSTM1* null (-/-) | 9/0 | **-** | **-** |
| *CYP1A1 m1* T/C + *CYP1A1 m2* A/A + *GSTM1* wild (+/+) | 38/36 | 1.08 (0.66, 1.78) | 0.74 |
| *CYP1A1 m1* T/C + *CYP1A1 m2* A/A + *GSTM1* null (-/-) | 6/15 | 0.39 (0.14, 1.02) | 0.04 |
| *CYP1A1 m1* T/C + *CYP1A1 m2* A/G + *GSTM1* wild (+/+) | 38/7 | **6.34** (2.77, 14.5) | **<0.001*** |
| *CYP1A1 m1* T/C + *CYP1A1 m2* A/G + *GSTM1* null (-/-) | 26/4 | **7.26** (2.49, 21.15) | **<0.001*** |
| *CYP1A1 m1* T/C + *CYP1A1 m2* G/G + *GSTM1* wild (+/+) | 11/1 | **11.66** (1.49, 90.9) | **<0.001***^#^ |
| *CYP1A1 m1* T/C + *CYP1A1 m2* G/G + *GSTM1* null (-/-) | 0/0 | - | **-** |
| *CYP1A1 m1* C/C + *CYP1A1 m2* A/A + *GSTM1* wild (+/+) | 3/11 | 0.33 (0.08, 1.26) | 0.16^#^ |
| *CYP1A1 m1* C/C + *CYP1A1 m2* A/A + *GSTM1* null (-/-) | 3/2 | 1.53 (0.25, 9.24) | 0.64^#^ |
| *CYP1A1 m1* C/C + *CYP1A1 m2* A/G + *GSTM1* wild (+/+) | 10/1 | **10.55** (1.34, 83.05) | **0.01***^#^ |
| *CYP1A1 m1* C/C + *CYP1A1 m2* A/G + *GSTM1* null (-/-) | 1/0 | - | - |
| *CYP1A1 m1* C/C + *CYP1A1 m2* G/G + *GSTM1* wild (+/+) | 5/0 | - | - |
| *CYP1A1 m1* C/C + *CYP1A1 m2* G/G + *GSTM1* null (-/-) | 1/0 | - | - |

***p<0.05; ^#^ yates corrected chi square**

**Table S5: Risk of NSCLC associated with combination of three genotypes (*CYP1A1m1*, *CYP1A1m2* and *GSTT1*)**

| **Genotype combination** | **Cases/ Controls** | **OR (95% CI)** | **p** |
| --- | --- | --- | --- |
| *CYP1A1 m1* T/T + *CYP1A1 m2* A/A + *GSTT1* wild (+/+) | 13/123 | 0.05 (0.03, 0.10) | <0.001 |
| *CYP1A1 m1* T/T + *CYP1A1 m2* A/A + *GSTT1* null (-/-) | 13/11 | 1.21 (0.53, 2.76) | 0.64 |
| *CYP1A1 m1* T/T + *CYP1A1 m2* A/G + *GSTT1* wild (+/+) | 62/13 | **6.14** (3.27, 11.51) | **<0.001*** |
| *CYP1A1 m1* T/T + *CYP1A1 m2* A/G + *GSTT1* null (-/-) | 3/7 | 0.42 (0.10, 1.67) | 0.35^#^ |
| *CYP1A1 m1* T/T + *CYP1A1 m2* G/G + *GSTT1* wild (+/+) | 9/19 | 0.46 (0.20, 1.04) | 0.05 |
| *CYP1A1 m1* T/T + *CYP1A1 m2* G/G + *GSTT1* null (-/-) | 5/0 | - | - |
| *CYP1A1 m1* T/C + *CYP1A1 m2* A/A + *GSTT1* wild (+/+) | 34/48 | 0.67 (0.41, 1.09) | 0.10 |
| *CYP1A1 m1* T/C + *CYP1A1 m2* A/A + *GSTT1* null (-/-) | 10/3 | **3.48** (0.94, 12.83) | **0.04***^#^ |
| *CYP1A1 m1* T/C + *CYP1A1 m2* A/G + *GSTT1* wild (+/+) | 51/8 | **7.91** (3.66, 17.07) | **<0.001*** |
| *CYP1A1 m1* T/C + *CYP1A1 m2* A/G + GSTTT1 null (-/-) | 13/3 | **4.59** (1.29, 16.32) | **0.02***^#^ |
| *CYP1A1 m1* T/C + *CYP1A1 m2* G/G + *GSTT1* wild (+/+) | 10/1 | **10.55** (1.34, 83.05) | **0.01***^#^ |
| *CYP1A1 m1* T/C + *CYP1A1 m2* G/G + *GSTT1* null (-/-) | 1/0 | - | - |
| *CYP1A1 m1* C/C + *CYP1A1 m2* A/A + *GSTT1* wild (+/+) | 2/9 | 0.21 (0.04, 1.02) | 0.07^#^ |
| *CYP1A1 m1* C/C + *CYP1A1 m2* A/A + *GSTT1* null (-/-) | 1/2 | 0.50 (0.04, 5.61) | 0.98^#^ |
| *CYP1A1 m1* C/C + *CYP1A1 m2* A/G + *GSTT1* wild (+/+) | 14/3 | **4.9** (1.41, 17.51) | **0.01***^#^ |
| *CYP1A1 m1* C/C + *CYP1A1 m2* A/G + *GSTT1* null (-/-) | 0/0 | - | - |
| *CYP1A1 m1* C/C + *CYP1A1 m2* G/G + *GSTT1* wild (+/+) | 14/0 | **-** | **-** |
| *CYP1A1 m1* C/C + *CYP1A1 m2* G/G + *GSTT1* null (-/-) | 0/0 | - | - |

***p<0.05; ^#^ yates corrected chi square**

**Table S6: Risk of NSCLC associated with combination of three genotypes (*CYP1A1m1*, *GSTM1* and *GSTT1*)**

| **Genotype combination** | **Cases/ Controls** | **OR (95% CI)** | **p** |
| --- | --- | --- | --- |
| ***CYP1A1 m1*** T/T + ***GSTM1* wild** (+/+) + ***GSTT1* wild** (+/+) | 57/118 | 0.33 (0.22, 0.49) | <0.001 |
| *CYP1A1 m1* T/T + *GSTM1* wild (+/+) +*GSTT1* null (-/-) | 17/15 | 1.16 (0.56, 2.38) | 0.68 |
| *CYP1A1 m1* T/T + *GSTM1* null (-/-) + *GSTT1* wild (+/+) | 27/37 | 0.70 (0.41, 1.20) | 0.20 |
| *CYP1A1 m1* T/T + *GSTM1* null (-/-) + *GSTT1* null (-/-) | 4/3 | 1.36 (0.30, 6.14) | 0.98^#^ |
| *CYP1A1 m1* T/C + *GSTM1* wild (+/+) + *GSTT1* wild (+/+) | 69/39 | **2.1** (1.35, 3.27) | **<0.001*** |
| *CYP1A1 m1* T/C + *GSTM1* wild (+/+) +*GSTT1* null (-/-) | 17/5 | **3.63** (1.32, 10.02) | **<0.001*** |
| *CYP1A1 m1* T/C + *GSTM1* null (-/-) + *GSTT1* wild (+/+) | 25/18 | 1.45 (0.77, 2.74) | 0.24 |
| *CYP1A1 m1* T/C + *GSTM1* null (-/-) + *GSTT1* null (-/-) | 6/1 | 6.22 (0.74, 52.08) | 0.05^#^ |
| *CYP1A1 m1* C/C + *GSTM1* wild (+/+) + *GSTT1* wild (+/+) | 20/8 | **2.67** (1.15, 6.19) | **0.01*** |
| *CYP1A1 m1* C/C + *GSTM1* wild (+/+) +*GSTT1* null (-/-) | 1/2 | 0.50 (0.04, 5.61) | 0.98^#^ |
| *CYP1A1 m1* C/C + *GSTM1* null (-/-) + *GSTT1* wild (+/+) | 1/4 | 0.25 (0.02, 2.26) | 0.37^#^ |
| *CYP1A1 m1* C/C + *GSTM1* null (-/-) + *GSTT1* null (-/-) | 0/0 | **-** | **-** |
| ***CYP1A1 m2*** A/A + ***GSTM1*** wild (+/+) + ***GSTT1* wild** (+/+) | 39/88 | 0.34 (0.22, 0.53) | <0.001 |
| *CYP1A1 m2* A/A + *GSTM1* wild (+/+) + *GSTT1* null (-/-) | 24/10 | **2.59** (1.21, 5.54) | **0.01*** |
| *CYP1A1 m2* A/A + *GSTM1* null (-/-) + *GSTT1* wild (+/+) | 10/50 | 0.16 (0.08, 0.34) | <0.01 |
| *CYP1A1 m2* A/A + *GSTM1* null (-/-) + *GSTT1* null (-/-) | 6/0 | **-** | **-** |
| *CYP1A1 m2* A/G + *GSTM1* wild (+/+) + *GSTT1* wild (+/+) | 88/15 | **1.97** (1.32, 2.93) | **<0.001*** |
| *CYP1A1 m2* A/G + *GSTM1* wild (+/+) +*GSTT1* null (-/-) | 10/10 | 1.01 (0.41, 2.48) | 0.97 |
| *CYP1A1 m2* A/G + *GSTM1* null (-/-) + *GSTT1* wild (+/+) | 39/9 | **5.04** (2.38, 10.66) | **<0.01*** |
| *CYP1A1 m2* A/G + *GSTM1* null (-/-) + *GSTT1* null (-/-) | 6/0 | **-** | **-** |
| *CYP1A1 m2* G/G + *GSTM1* wild (+/+) + *GSTT1* wild (+/+) | 19/20 | 0.96 (0.50, 1.85) | 0.90 |
| *CYP1A1 m2* G/G + *GSTM1* wild (+/+) +*GSTT1* null (-/-) | 2/0 | - | - |
| *CYP1A1 m2* G/G + *GSTM1* null (-/-) + *GSTT1* wild (+/+) | 5/0 | - | - |
| *CYP1A1 m2* G/G + *GSTM1* null (-/-) + *GSTT1* null (-/-) | 3/0 | - | - |

***p<0.05; ^#^ yates corrected chi square**

**Table S7: Risk of NSCLC associated with combination of four genotypes (*CYP1A1m1*, *CYP1A1m2*, *GSTM1* and *GSTT1*)**

| **Genotype combination** | **Cases/ Controls** | **OR (95% CI)** | **p** |
| --- | --- | --- | --- |
| *CYP1A1 m1* T/T + *CYP1A1 m2* A/A + *GSTM1* wild (+/+) + *GSTT1* wild (+/+) | 9/92 | 0.06 (0.03, 0.13) | 0.01 |
| *CYP1A1 m1* T/T + *CYP1A1 m2* A/A + *GSTM1* wild (+/+) + *GSTT1* null (-/-) | 13/8 | 1.68 (0.68, 4.14) | 0.25 |
| *CYP1A1 m1* T/T + *CYP1A1 m2* A/A + *GSTM1* null (-/-) + *GSTT1* wild (+/+) | 4/34 | 0.10 (0.03, 0.30) | <0.01 |
| *CYP1A1 m1* T/T + *CYP1A1 m2* A/A + *GSTM1* null (-/-) + *GSTT1* null (-/-) | 0/0 | _ |  |
| *CYP1A1 m1* T/T + *CYP1A1 m2* A/G + *GSTM1* wild (+/+) + *GSTT1* wild (+/+) | 44/10 | **5.22** (2.56, 10.65) | **<0.001*** |
| *CYP1A1 m1* T/T + *CYP1A1 m2* A/G + *GSTM1* wild (+/+) + *GSTT1* null (-/-) | 3/7 | 0.42 (0.10, 1.67) | 0.35^#^ |
| *CYP1A1 m1* T/T + *CYP1A1 m2* A/G + *GSTM1* null (-/-) + *GSTT1* wild (+/+) | 18/3 | **6.5** (1.89, 22.34) | **<0.001***^#^ |
| *CYP1A1 m1* T/T + *CYP1A1 m2* A/G + *GSTM1* null (-/-) + *GSTT1* null (-/-) | 0/0 | _ |  |
| *CYP1A1 m1* T/T + *CYP1A1 m2* G/G + *GSTM1* wild (+/+) + *GSTT1* wild (+/+) | 4/19 | 1.61 (0.68, 3.80) | 0.26^#^ |
| *CYP1A1 m1* T/T + *CYP1A1 m2* G/G + *GSTM1* wild (+/+) + *GSTT1* null (-/-) | 1/0 | - | - |
| *CYP1A1 m1* T/T + *CYP1A1 m2* G/G + *GSTM1* null (-/-) + *GSTT1* wild (+/+) | 5/0 | - | - |
| *CYP1A1 m1* T/T + *CYP1A1 m2* G/G + *GSTM1* null (-/-) + *GSTT1* null (-/-) | 3/0 | - | - |

***p<0.05; ^#^ yates corrected chi square**

**Table S8: Risk of lung NSCLC associated with combination of four genotypes (*CYP1A1m1*, *CYP1A1m2*, *GSTM1* and *GSTT1*)**

| **Genotype combination** | **Cases/ Controls** | **OR (95% CI)** | **p** |
| --- | --- | --- | --- |
| *CYP1A1 m1* T/C + *CYP1A1 m2* A/A + *GSTM1* wild (+/+) + *GSTT1* wild (+/+) | 28/35 | 0.78 (0.46, 1.34) | 0.38 |
| *CYP1A1 m1* T/C + *CYP1A1 m2* A/A + *GSTM1* wild (+/+) + *GSTT1* null (-/-) | 10/2 | **5.25** (1.13, 24.23) | **0.01***^#^ |
| *CYP1A1 m1* T/C + *CYP1A1 m2* A/A + *GSTM1* null (-/-) + *GSTT1* wild (+/+) | 6/14 | 0.42 (0.15, 1.11) | 0.07 |
| *CYP1A1 m1* T/C + *CYP1A1 m2* A/A + *GSTM1* null (-/-) + *GSTT1* null (-/-) | 0/0 | _ |  |
| *CYP1A1 m1* T/C + *CYP1A1 m2* A/G + *GSTM1* wild (+/+) + *GSTT1* wild (+/+) | 31/4 | **8.86** (3.08, 25.52) | **<0.001***^#^ |
| *CYP1A1 m1* T/C + *CYP1A1 m2* A/G + *GSTM1* wild (+/+) + *GSTT1* null (-/-) | 7/3 | 2.41 (0.61, 9.43) | 0.32^#^ |
| *CYP1A1 m1* T/C + *CYP1A1 m2* A/G + *GSTM1* null (-/-) + *GSTT1* wild (+/+) | 20/3 | **7.2** (2.13, 24.85) | **<0.001***^#^ |
| *CYP1A1 m1* T/C + *CYP1A1 m2* A/G + *GSTM1* null (-/-) + *GSTT1* null (-/-) | 6/0 | **-** | **-** |
| *CYP1A1 m1* T/C + *CYP1A1 m2* G/G + *GSTM1* wild (+/+) + *GSTT1* wild (+/+) | 10/0 | **-** | **-** |
| *CYP1A1 m1* T/C + *CYP1A1 m2* G/G + *GSTM1* wild (+/+) + *GSTT1* null (-/-) | 1/0 | - | - |
| *CYP1A1 m1* T/C + *CYP1A1 m2* G/G + *GSTM1* null (-/-) + *GSTT1* wild (+/+) | 0/0 | **-** | **-** |
| *CYP1A1 m1* T/C + *CYP1A1 m2* G/G + *GSTM1* null (-/-) + *GSTT1* null (-/-) | 0/0 | **-** | **-** |

***p<0.05; ^#^ yates corrected chi square**

**Table S9: Risk of NSCLC associated with combination of four genotypes (*CYP1A1m1*, *CYP1A1m2*, *GSTM1* and *GSTT1*)**

| **Genotype combination** | **Cases/ Controls** | **OR (95% CI)** | **p** |
| --- | --- | --- | --- |
| *CYP1A1 m1* C/C + *CYP1A1 m2* A/A + *GSTM1* wild (+/+) + *GSTT1* wild (+/+) | 2/9 | 0.21 (0.04, 1.02) | 0.07 |
| *CYP1A1 m1* C/C + *CYP1A1 m2* A/A + *GSTM1* wild (+/+) + *GSTT1* null (-/-) | 1/2 | 0.50 (0.04, 5.61) | 0.98^#^ |
| *CYP1A1 m1* C/C + *CYP1A1 m2* A/A + *GSTM1* null (-/-) + *GSTT1* wild (+/+) | 0/0 | - | **-** |
| *CYP1A1 m1* C/C + *CYP1A1 m2* A/A + *GSTM1* null (-/-) + *GSTT1* null (-/-) | 0/0 | - | **-** |
| *CYP1A1 m1* C/C + *CYP1A1 m2* A/G + *GSTM1* wild (+/+) + *GSTT1* wild (+/+) | 13/1 | **13.89** (1.80, 107.0) | **<0.001***^#^ |
| *CYP1A1 m1* C/C + *CYP1A1 m2* A/G + *GSTM1* wild (+/+) + *GSTT1* null (-/-) | 0/0 | - | **-** |
| *CYP1A1 m1* C/C + *CYP1A1 m2* A/G + *GSTM1* null (-/-) + *GSTT1* wild (+/+) | 1/2 | 0.50 (0.04, 5.61) | 0.98^#^ |
| *CYP1A1 m1* C/C + *CYP1A1 m2* A/G + *GSTM1* null (-/-) + *GSTT1* null (-/-) | 0/0 | - | - |
| *CYP1A1 m1* C/C + *CYP1A1 m2* G/G + *GSTM1* wild (+/+) + *GSTT1* wild (+/+) | 4/0 | - | - |
| *CYP1A1 m1* C/C + *CYP1A1 m2* G/G + *GSTM1* wild (+/+) + *GSTT1* null (-/-) | 0/0 | - | **-** |
| *CYP1A1 m1* C/C + *CYP1A1 m2* G/G + *GSTM1* null (-/-) + *GSTT1* wild (+/+) | 0/0 | - | **-** |
| *CYP1A1 m1* C/C + *CYP1A1 m2* G/G + *GSTM1* null (-/-) + *GSTT1* null (-/-) | 0/0 | - | **-** |

***p<0.05; ^#^ yates corrected chi square**
